# Salicylic Acid in Root Growth and Development

**DOI:** 10.3390/ijms23042228

**Published:** 2022-02-17

**Authors:** Zulfira Z. Bagautdinova, Nadya Omelyanchuk, Aleksandr V. Tyapkin, Vasilina V. Kovrizhnykh, Viktoriya V. Lavrekha, Elena V. Zemlyanskaya

**Affiliations:** 1 Institute of Cytology and Genetics, Siberian Branch, Russian Academy of Sciences, 630090 Novosibirsk, Russia; zulfira@bionet.nsc.ru (Z.Z.B.); nadya@bionet.nsc.ru (N.O.); surtur1001@yandex.ru (A.V.T.); vasilinakovr@gmail.com (V.V.K.); vvl@bionet.nsc.ru (V.V.L.); 2 Department of Natural Sciences, Novosibirsk State University, 630090 Novosibirsk, Russia

**Keywords:** salicylic acid, root growth, lateral roots, adventitious roots, abiotic stress, plant defense, auxin

## Abstract

In plants, salicylic acid (SA) is a hormone that mediates a plant’s defense against pathogens. SA also takes an active role in a plant’s response to various abiotic stresses, including chilling, drought, salinity, and heavy metals. In addition, in recent years, numerous studies have confirmed the important role of SA in plant morphogenesis. In this review, we summarize data on changes in root morphology following SA treatments under both normal and stress conditions. Finally, we provide evidence for the role of SA in maintaining the balance between stress responses and morphogenesis in plant development, and also for the presence of SA crosstalk with other plant hormones during this process.

## 1. Introduction

Recognized as the sixth plant hormone in 1992 [1], 2-hydroxybenzoic or salicylic acid (SA) belongs to a family of naturally occurring phenolic compounds which possess an aromatic benzene ring bearing one or more hydroxyl groups. Since then, a huge amount of data has been accumulated on SA’s involvement in various biological processes. As reviewed in [2,3], SA has secured a reputation as a vital defense hormone. At the same time, SA’s impact on cell, tissue, and organ phenotypes is well established (reviewed in [4]). Despite the growing evidence that SA is an important growth regulator, its morphogenetic role, especially in relation to roots, has rarely been summarized in reviews.

It is worthy of note that, in roots, SA content and its dynamic during development may differ from that in shoots [5,6,7,8], which can potentially cause differences in SA functions. For instance, SA basal level in shoots is 2–100 times higher than in roots, depending on the species [5,6]. The ratio between free and conjugated SA forms, also differs [6]. For example, the shoots of wheat seedlings, three days after germination (DAG), contain about 48 times more free than conjugated SA, whereas in the roots, the contrary is seen, with conjugated SA levels exceeding the level of free SA by about six times. During wheat seedling growth, SA content in both free and conjugated forms gradually decreases in shoots but not in roots. In 14 DAG seedlings, the conjugated SA becomes prevalent in both organs but the ratio of free to conjugated form still differs slightly and amounts to 0.4 and 0.5 for shoots and roots, respectively. These differences provide ample reason to consider the role of SA in root morphology, distinct from its function in shoots.

The phenotypic analysis of SA deficient/accumulating lines and SA-treated plants provides insight into the role of SA in plant growth and development. However, the data on changes in root morphology in SA mutants are scarce and often contradictory. For example, decreased root length is reported in SA-accumulating Arabidopsis mutants [9,10,11] and in SA-depleted rice mutants [12,13]. In rice, the inhibitory effects of an SA deficiency on root length have been reported in relation to both SA biosynthesis mutant *aim1* [12] and plants transgenic for the bacterial *Naphthalene hydroxylase G* (*NahG*) gene, encoding salicylate hydroxylase that inactivates SA by converting it to catechol [13]. In contrast, transgenic *Lotus japonicus* plants expressing *NahG*, demonstrate enhanced root growth [14]. These contradictions may be due to species-specific basal SA levels, which vary greatly between plant species, even those belonging to the same family [4,15].

In contrast to SA mutants, changes in root morphology after an SA treatment are described in numerous papers. In our review, we analyzed more than 100 studies on SA treatments in 40 plant species, with SA doses ranging from 10 fM to 10 mM, to survey the impact of SA on root system biomass and architecture under normal conditions and in response to stressors. In this paper, we discuss various aspects of SA’s regulation of root growth and development, including changes in root morphology after SA treatments, the molecular basis of these changes, and the impact of SA in root responses to abiotic stress and other environmental changes.

## 2. SA Metabolism and Signaling in Plants

**Figure 1:**
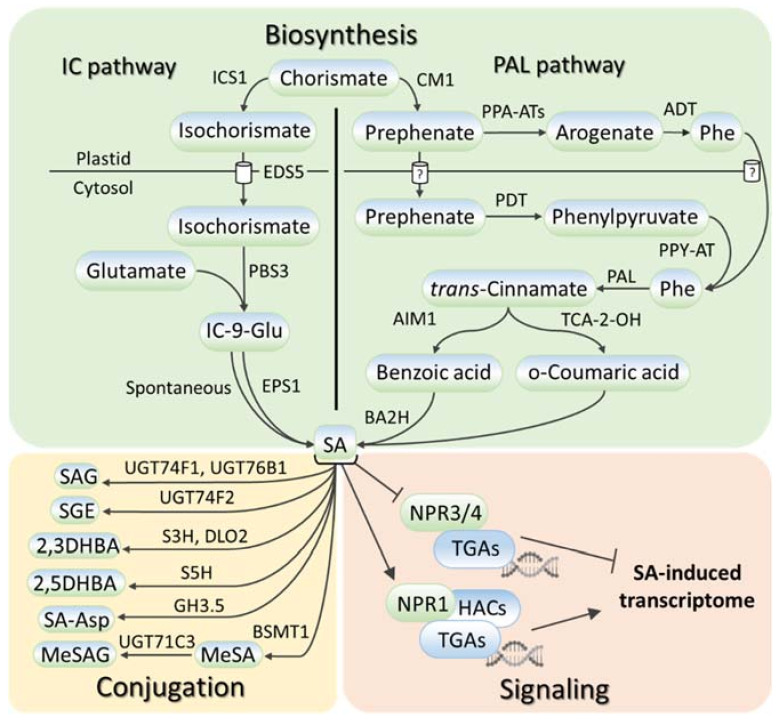
SA metabolism and signaling in plants. SA is synthesized via two routes, the isochorismate pathway or the phenylalanine ammonia-lyase pathway, which both start with chorismate. SA conversions include SA glycosylation, methylation, hydroxylation, and amino-acid conjugation. SA signaling depends on the interaction of SA receptor NPR1 with TGA transcription factors and histone acetyltransferases. SA, salicylic acid; ICS1, isochorismate synthase 1; EDS5, ENHANCED DISEASE SUSCEPTIBILITY 5; PBS3, avrPphB Susceptible 3; EPS1, Enhanced Pseudomonas Susceptibility 1; IC-9-Glu, isochorismate-9-glutamate; CM1, chorismate mutase 1; PPA-ATs, prephenate aminotransferases; PDT, prephenate dehydratase; PPY-AT, phenylpyruvate aminotransferase; ADT, arogenate dehydratase; PAL, phenylalanine ammonia-lyase; AIM1, Abnormal Inflorescence Meristem 1; TCA-2-OH, trans-cinnamic acid 2-hydroxylase; BA2H, benzoic acid 2-hydroxylase; UGT74F1/74F2/76B1/71C3, UDP-glucosyltransferases 74F1, 74F2, 76B1 and 71C3; S5H, SA-5 hydroxylase; S3H, SA-3 hydroxylase; DLO2, DMR6-LIKE OXYGENASE 2; GH3.5, Gretchen Hagen 3.5; BSMT1, benzoic acid/salicylic acid methyltransferase; SAG, salicylic acid 2-*O*-β-D-glucose; SGE, salicylic acid glucose ester; 2,3/2,5-DHBA, 2,3/2,5-dihydroxybenzoic acid; SA-Asp, salicyloyl-L-aspartate; MeSA, methyl salicylate; MeSAG, methyl salicylate O-β-glucoside; NPR1/3/4, NONEXPRESSOR OF PATHOGENESIS RELATED GENES 1/3/4; HACs, histone acetyltransferases; TGA, TGACG SEQUENCE-SPECIFIC BINDING PROTEIN.

SA metabolism has been comprehensively described in numerous reviews, for example, [16,17,18,19,20]; therefore, we touch only briefly on this aspect in this review. SA is synthesized in plants, bacteria, and fungi from chorismate, the final product in the shikimate pathway (reviewed in [16]). Chorismate is also the primary source for the biosynthesis of aromatic amino acids (tryptophan, phenylalanine, and tyrosine) and a wide range of aromatic secondary metabolites, including flavonoids, alkaloids, and lignins. SA biosynthesis (Figure 1, Table S1) starts in plastids, where chorismate is converted into either isochorismate via isochorismate synthase (ICS) or prephenate via chorismate mutase (CM), giving rise to two parallel ICS and phenylalanine ammonia-lyase (PAL) pathways of SA biosynthesis (reviewed in [16,17,19,20]). The relative contributions of the ICS and PAL pathways to SA biosynthesis are species-dependent with an equal contribution being made in soybean and a prevalence of ICS and PAL pathways being seen in Arabidopsis and rice, respectively. In the ICS pathway, ENHANCED DISEASE SUSCEPTIBILITY 5 (EDS5) transports isochorismate to the cytosol, where it is conjugated with glutamate by avrPphB Susceptible 3 (PBS3) to produce isochorismate-9-glutamate, which is either spontaneously decomposed into SA or converted to SA by an acyltransferase Enhanced Pseudomonas Susceptibility 1 (EPS1). In the PAL pathway, there are two ways of prephenate elaboration into phenylalanine (reviewed in [18]). In plastids, prephenate aminotransferases (PPA-ATs) catalyze its transition to arogenate, which is then converted by arogenate dehydratase (ADT) into phenylalanine. In the cytosol, the prephenate–phenylalanine transition is realized through phenylpyruvate by prephenate dehydratase (PDT) and phenylpyruvate aminotransferase (PPY-AT). PAL turns phenylalanine into trans-cinnamic acid, following which the formation of SA occurs either through ortho-coumaric acid or benzoic acid (reviewed in [16,17,19,20]). In the latter case, Abnormal Inflorescence Meristem 1 (AIM1), a 3-hydroxyacyl-CoA dehydrogenase, contributes to this process. Benzoic acid is hydroxylated to SA, possibly by benzoic acid 2-hydroxylase (BA2H).

A wide number of regulators control SA biosynthesis (Table S1). Among them, the reactive oxygen species (ROS), particularly hydrogen peroxide, form a self-amplifying feedback loop with SA, in which hydrogen peroxide promotes SA biosynthesis, and SA induces hydrogen peroxide accumulation by inactivating its scavengers [21,22] (reviewed in [23]).

SA levels are regulated not only by SA biosynthesis but also by SA chemical modifications and intercellular transport (reviewed in [18,24]). These processes have been studied mainly in Arabidopsis. SA glycosylation occurs via the conjugation of glycosyl onto the hydroxyl and carboxyl groups of SA, producing two inactive SA storage forms, salicylic acid 2-*O-β*-d-glucose (SAG) and salicylic acid glucose ester (SGE). Uridine diphosphate (UDP)-glycosyltransferases UGT74F1 and UGT74F2 perform the conversion to the former, while only UGT74F2 is involved in the catalysis to the latter. The carboxyl group can be also methylated by the S-adenosyl-L-methionine (SAM)-dependent methyltransferase, BA/SA carboxyl methyltransferase 1 (BSMT1), producing methyl salicylate (MeSA), the form of SA that has increased membrane permeability. SA hydroxylation by SA-5 and SA-3 hydroxylases generates 2,3-DHBA and 2,5-DHBA dihydroxybenzoic acids. Gretchen Hagen 3.5/WESO 1 (GH3.5/WES1) and another unknown GH3 family enzyme convert SA into salicyloyl-L-aspartate (SA-Asp). Some of these conjugated forms of SA may also be glycosylated. Inactive SA forms can be stored until they are required to increase the active pool of free SA; alternatively, some of them may be subjected to SA catabolism. SA is often spread via apoplast (reviewed in [24]). Since SA is a weak acid with poor water solubility, the existence of influx and efflux carriers along with pH-dependent diffusion is proposed for its movement through the plasma membrane.

The NONEXPRESSOR OF PATHOGENESIS-RELATED GENES (NPR) are the SA receptors (reviewed in [3,18,24]). At a low SA level, NPR1 oligomerizes in the cytosol. Meanwhile, NPR1 paralogs, NPR3 and NPR4, directly interact with the basic leucine zipper (bZIP) family’s TGA transcription factors on the promoters of NPR1 targets, to suppress their expression. SA facilitates the reduction of cytosolic NPR1 oligomers into monomers, which are translocated to the nucleus and activate transcription in complex with TGAs. At the same time, SA inhibits the activity of NPR3 and NPR4 to allow for the transcription of SA-responsive genes. The NPR1 pathway is functional in both shoots and roots [25,26]. SA also binds to A subunits of protein phosphatase 2A (PP2A) and inhibits the activity of this enzyme, thereby altering auxin transport and distribution [26]. There are other SA binding proteins as well but their functions in SA signaling are largely unknown [27,28,29,30] (reviewed in [31,32]).

## 3. Modulation of Endogenous SA Levels in Roots

In Arabidopsis shoots, the basal level of SA amounts to 0.25–1 µg per gram of the fresh weight, rising up to 20 µg.g^−1^ at the place of pathogen attack [33] (reviewed in [4]). In many plant species roots also accumulate SA upon invasion of soil-borne pathogens (Table 1). Rapid SA accumulation is a part of plant immune signaling, which has been extensively studied in shoots. In this process, SA promotes pathogen-associated molecular pattern (PAMP)-triggered immunity (PTI), effector-triggered immunity (ETI), and systemic acquired resistance (SAR), via an NPR-dependent activation of plant defense genes, to resist biotrophic and semi-biotrophic pathogens (reviewed in [2,3,34]). The mechanisms of plant defense in roots are less studied, yet pathogen-induced SA accumulation is considered an essential factor in root protection from biotic stress [35] (reviewed in [36]). The attacks of soil-borne pathogens are capable of inducing systemic SA accumulation in above-ground tissue [37], and mutants and transgenic plants with a reduced ability to accumulate SA are more susceptible to root infections than wild types [38,39,40,41].

SA can also accumulate in roots in response to abiotic stresses such as aluminum, cadmium, chilling, salt, drought, UV-B radiation exposure, alkalinity, and iron- and nitrogen deficiency (Table 1), echoing the reported role of SA in abiotic stress resistance (reviewed in [57,58]). In some cases, stress-induced SA accumulates locally in the root, which comes into direct contact with the stress factor, but it can also be transported to the aboveground tissue. For example, in barley, SA accumulates in response to drought in the roots but not in shoots [51]. In grapes exposed to heat stress, SA is progressively transported from the roots to shoots via xylem [59]. Stress-induced changes in endogenous SA levels are species-specific. For example, drought increases SA content in barley [51] but reduces it in *Scutellaria baicalensis* roots [53].

SA (50 μM) treatment promotes adventitious root development at the base of cucumber hypocotyls and strongly increases endogenous SA levels in the rooting zone [60]. It is worthy of note that an SA treatment does not necessarily elevate endogenous SA levels in the root due to exogenous SA uptake. For example, priming wheat seed with SA (50 µM, 3 h) or treating 10 DAG seedlings with 500 µM SA for 1–24 h reduces the endogenous levels of both free and conjugated SA in roots [6,47]. The endogenous levels of total and free SA in *Scutellaria baicalensis* roots also decrease when seedlings are treated with 300 µM SA [53]. Therefore, the type and intensity of exogenous SA effects on plant growth are probably related to changes in the plant’s endogenic SA content and/or redistribution of free and conjugated forms. Accordingly, a feasible role of SA biosynthesis in endogenous SA content after an exogenous SA treatment was demonstrated in several studies [61,62]. Priming maize seeds with [3,4,5,6-2 H4]-salicylic acid (D4SA; the SA deuterated isotopomer) during germination allowed researchers to estimate both SA uptake and SA’s regulation of its own biosynthesis in developing roots [63]. A low SA concentration (50 µM) increased both SA uptake and biosynthesis, whereas a high SA level (500 µM) more strongly enhanced SA uptake but inhibited SA biosynthesis.

Growing evidence indicates that normal plant growth requires that optimal levels of endogenous SA are maintained. Accordingly, a number of negative regulators that alleviate SA accumulation (such as *CPR5*, *DND1*, *PI4KIIIꞵ1*, *PI4KIIIꞵ2* etc.) were described in relation to Arabidopsis [7,8,64,65]. Moreover, hybrids between Arabidopsis accessions with suboptimal and supraoptimal SA content (Columbia and C24, respectively) show root growth heterosis [66]. The chromatin remodeler DECREASED DNA METHYLATION 1 (DDM1) links heterosis with endogenous SA levels. Columbia/C24 hybrid heterosis in the root length is impaired in the *ddm1* mutant background.

## 4. SA Regulates Root Morphology in a Concentration-Dependent Manner

### 4.1. Regulation of Radicle Emergence

Seed germination proceeds as a transition from dormancy to the radicle (primary root) emergence, starting from water uptake by dry seeds (imbibition) and being completed with testa (seed coat) rupture and the radicle protrusion (reviewed in [67]). The primary root growth along with seed coat and endosperm weakening are three players in the completion of germination. The radicle growth is based on cell elongation in the hypocotyl-radicle transition zone [68].

A concentration-dependent effect of SA priming on seed germination, namely, an increased germination percent at low SA concentrations and a decreased percent at higher levels, was reported in experiments on carrots, cucumbers, and wheat [69,70,71,72] (Table 2). The activating/inhibitory SA concentrations are species-specific and amount to 7 μM/7 mM and 10–50 µM/100–500 µM in carrot and cucumber, respectively [69,70]. In wheat, the activating/inhibitory SA concentrations are also cultivar-specific and were 10–20 μM/30 μM and 500 µM/1 mM for two different cultivars [71,72]. In Arabidopsis, 100 µM SA enhances seed germination [73], whereas higher concentrations (250 μM–5 mM) retard it [74,75]. The SA concentration-dependent effect was also demonstrated in immature maize embryos. SA (0.5–1.5 mM) stimulated germination of isolated maize embryos at 25 days post pollination (DPP), whereas higher SA doses (3–5 mM) completely inhibited this process [76]. Older embryos (28 DPP) were stimulated by SA at a wider range of concentrations (0.5–3 mM).

For many species, SA concentrations that only activate or only inhibit germination have been described so far (Table S2). SA increases the percentage of germinated seeds in fenugreek (10 μM), *Limonium bicolor* (80–280 µM), black cumin (200–500 μM), rice (700 µM), and *Bromus tomentellus* (1.5–2 mM) [77,78,79,80,81,82]. It suppresses seed germination in sesame (350 μM), pearl millet (500 µM), lentil and barley (1 mM), *Lactuca sativa*, *Deschampsia flexuosa*, and *Chamaenerion angustifolium* (10 mM) [83].

Other hormones also participate in primary root emergence. Only promoting effects are reported for ethylene precursor 1-aminocyclopropane-1-carboxylic acid (ACC) and brassinosteroids, and only inhibitory effects for jasmonic (JA) and abscisic (ABA) acids and auxin [84,85,86,87,88,89,90,91,92,93,94,95,96,97,98,99,100,101,102,103,104,105,106,107]. ABA suppresses radicle emergence by inhibiting cell wall loosening and cell elongation [108]. ABA extrusion from the hypocotyl-radicle transition zone takes place before radicle protrusion [109]. Auxin delays rupture of the soybean testa and radicle emergence by stimulating ABA biosynthesis and impairing gibberellins (GAs) biosynthesis [104]. JA activates ABA signaling [110]. Cytokinins and GAs mainly have a stimulating effect on seed germination [91,104,105,106,107,111,112,113], wherein GAs promote germination activating SA biosynthesis and signaling [114], and cytokinin antagonizes ABA-mediated inhibition of germination by suppressing *ABA INSENSITIVE5 (ABI5)* [115]. However, in some species, inhibition of radicle emergence upon cytokinin and GAs treatment has been reported [71,116]. GAs and brassinosteroids also increase cell elongation in the hypocotyl-radicle transition zone [117,118].

### 4.2. SA Impact on Root Length

A concentration-dependent effect of SA on the radicle/primary root length in dicots was shown for fenugreek, cucumber, lentil, and bean [70,77,119,120] (Table 3) when low SA concentrations stimulated and higher ones retarded root growth. The activating/inhibitory SA concentrations differ in different species and amount to 5–10 μM/15 μM; 10–50 µM/100–500 µM; 100–500 µM/1 mM; and 500 µM/1 mM for fenugreek, cucumber, lentil, and bean, respectively. In monocots, the concentration-dependent influence of SA on radicle growth was found in wheat where, similar to dicots, low (10 μM) and high (30 μM) SA levels increased and decreased radicle length, respectively [71]. In other papers on SA priming of wheat seeds, only an increase in the length of radicle or seminal roots were reported despite the application of higher SA concentrations (50 μM–1 mM) [6,72,121,122] (Table S2). SA priming of pearl millet seeds from four varieties uncovers a strong influence of genotype on the root growth response to an SA treatment [123]. Thus, an SA treatment of 0.5 mM results in an increased root length in one variety, while SA applied in a concentration range of 0.5–3 mM decreases root length in another one. In two other varieties, a decrease in root length was obtained only at high (2–3 mM) SA concentrations. The genotype specificity of SA effective concentrations may explain the wide ranges reported to increase root length in tomato (0.1–100 μM) and rice (700 µM–1 mM) and to decrease root length in Arabidopsis (3–250 µM) [9,25,26,56,73,80,81,124,125,126,127,128,129,130]. In most species, there are only a few papers reporting only decreases or only increases in root length after an SA treatment (Table S2) and similar SA doses may enhance (1 µM–5 mM) or suppress (0.7 µM–10 mM) root growth depending on the species [42,79,83,97,131,132,133,134,135,136,137,138,139,140,141,142].

SA controls root growth by regulating root apical meristem (RAM) activity [12,25,143]. It has been shown that 30 µM SA reduces the number of cells expressing cell division marker CYCLIN B1;1 (CYCB1;1) in the proximal meristem [25]. The suppression of cell divisions in the proximal meristem explains the decreased root length after treatment with SA. A treatment with higher SA concentrations (150 µM) results in enlarged proximal meristem cells without any CYCB1;1 signal in more than a half of treated roots. In the rice *aim1* mutant, the contrary occurs. SA deficiency results in a decreased expression of several *CYCLIN* genes, a reduced RAM size, and the decreased longitudinal length of mature cells [12]. An SA treatment of 500 µM restores the activity of *CYCLIN* genes and makes the RAM size and longitudinal length of mature cells in the *aim1* mutant similar to those in SA-treated wild-type plants. SA inhibits the expression of genes related to redox homeostasis and ROS scavenging to maintain the ROS accumulation necessary for RAM activity. SA provides this partially through the induction of transcriptional repressors WRKY62 and WRKY76.

SA regulates root growth along with other plant hormones. Concentration-dependent effects on root growth were shown also for auxin (indole-3-acetic acid, IAA), ABA, and brassinosteroids [144,145,146,147,148,149]. In maize, low doses of IAA or ABA stimulate root growth only in seedlings with fast-growing roots and inhibit root growth in seedlings with slow-growing roots, indicating that endogenous hormone levels may not only determine the growth rate but also the manner of its modification in response to hormonal treatments [147]. In Arabidopsis, treatment with auxin (50–100 pM; both IAA and 2,4-D) increases root growth, which is inhibited at auxin concentrations above 1 nM [144,145,146,150]. This inhibition occurs via an extremely rapid non-transcriptionally regulated adaptation of the root growth rate to the auxin level, which suggests that free and ubiquitinated Auxin/INDOLE-3-ACETIC ACID (Aux/IAA) proteins promote and inhibit root growth, respectively [150]. The formation of the TRANSPORT INHIBITOR RESPONSE 1/AUXIN SIGNALING F-BOX PROTEIN–Aux/IAA (TIR1/AFB–Aux/IAA) complex is required for this rapid root growth regulation. We suggest that SA is one of the interactors within this complex. In the auxin signaling pathway, SA reduces TIR1 and AFB1 receptor levels, resulting in the stabilization of Aux/IAA proteins, which inhibits the auxin response [151]. SA (1 mM) triggers the repression of TIR1 receptors [152]. In addition, endogenous auxin controls apoplast acidification and alkalization, which are required for the activation or repression of root cell elongation, respectively [153]. For ethylene, cytokinin, and jasmonic acid only inhibitory effects on root growth were demonstrated, while for gibberellins only stimulating effects on root growth were shown in various species [89,106,113,116,154,155,156,157,158,159,160,161,162,163,164,165,166,167,168,169,170,171,172,173,174,175,176,177,178,179,180,181,182,183]. SA crosstalks with cytokinin in root growth regulation [166]. In Arabidopsis, the cytokinin, benzyl adenine (BA), inhibits primary root growth in the wild type at 50 nM, whereas, in the *eds16* mutant, which is deficient in SA biosynthesis, this occurs at a lower (5 nM) concentration.

### 4.3. SA Regulates the Development of Lateral Roots

In Arabidopsis, predetermination of the lateral root founder cells occurs in a subset of xylem pole pericycle cells, followed by several anticlinal divisions, and culminates with cell elongation (reviewed in [184]). In the transition zone, oscillations in auxin signaling, within a period of about 6 h, marks the founder cell; this oscillatory pattern persists in the elongation zone [185]. In the differentiation zone, the founder cell becomes the prebranch site, with sustained elevated levels of auxin, and founder cell specification precedes lateral root initiation, which is marked by asymmetric founder cell division (reviewed in [184]). Lateral root primordium formation with establishment of a new root apical meristem and lateral root emergence are the final steps of lateral root development. There are some species-specific traits in lateral root development. For example, in maize, lateral roots originate in the phloem pole pericycle cells, and in rice, both pericycle and endodermal cells contribute to this process [186] (reviewed in [187]).

Adding SA (3–250 µM) to the growth medium reduces the number of lateral roots and lateral root primordia in Arabidopsis seedlings [9,25,26,128,188] (Table S2). This SA-induced phenotype is related to plant defense (see below). In contrast, in plant tissue cultures of the *Catharanthus roseus* hairy root line obtained from *Agrobacterium rhizogenes* infected leaves, SA at a very low concentration (10 fM) increases lateral root number, causes their early emergence (closer to the root tip), and enhances their growth [189]. However, before a firm conclusion as to the impact of different SA doses in lateral root development can be arrived at, further investigation in other species, at low SA concentrations, is needed.

In Arabidopsis, IAA promotes lateral root initiation at low concentrations (1–5 nM) and inhibits it at higher concentrations (25 nM and more) [190]. In addition, the influence of auxin on lateral root development depends on the lateral root developmental stage [190,191]. The impact of ethylene on lateral root formation is also dose-dependent [190,192]. ABA (100 nM–10 µM) stimulates lateral root formation in most legume species (both nodulating and not nodulating) and suppresses this process in nonlegume species [193]. In *Populus*, gibberellin applied directly to the shoot apex inhibits lateral root initiation via crosstalk with auxin and ABA [194]. On the other hand, in Arabidopsis, GA3 (10 µM) increases the number of lateral root primordia [128]. Brassinosteroids and jasmonates activate, whereas cytokinins inhibit, lateral root formation [102,128,165,167,169,172].

### 4.4. SA Regulates the Development of Adventitious Roots

The dose-dependent effects of SA on adventitious rooting is described in relation to three species: Arabidopsis, azalea, and mung beans, where low SA concentrations (3–50 µM; 100 µM; and 200–600 µM, respectively) increase the percentage of plants with adventitious roots or the number of adventitious roots, and higher concentrations (100–200 µM; 10 mM; and 800 µM, respectively) decrease these parameters [25,142,195] (Table 4). In adventitious root cultures of madder, SA (20 µM) enhances root growth and elevates fresh and dry root weights [133] (Table S2).

In cucumber, SA (50–100 μM) competitively inhibits the formation of IAA-Asp by CsGH3.5, increases free IAA level, and promotes the formation of adventitious roots [60]. IAA ( 10 µM) increases the number of adventitious roots per explant more than SA (50 µM) but decreases the average length of adventitious roots less than SA. Combined IAA and SA treatments enhance the action of both hormones in respect of root length and reproduce the IAA result in terms of the number of roots. Auxin also increases the endogenous SA level in adventitious rooting [196]. In carnation stem cuttings, auxin initiates an SA increase just after treatment, and SA levels peak at 12 h. Adventitious rooting of stem slices from apple microshoots moves through three stages: dedifferentiation, induction, and differentiation with an outgrowth [197]. SA enhances IAA decay via decarboxylation and because of this SA (30 µM) inhibits the initial stages of rooting (0–120 h) that is promoted by auxin, and enhances the stage of root outgrowth, which is suppressed by auxin [198]. SA has the opposite effects on rooting depending on the IAA concentration in slice treatments with both hormones. At 30 µM and 100 µM IAA, the application of 30 µM SA reduces and elevates the number of adventitious roots, respectively. Along with IAA and SA, GH3.5 and its paralogs GH3.3 and GH3.6 conjugate JA and thereby fine-tune adventitious rooting [60,199,200]. Treatment with jasmonates or cytokinins inhibits adventitious root initiation [201,202]. Similar to SA, brassinosteroids influence adventitious rooting in a concentration-dependent manner, promoting rooting at low concentrations (1 µM) and inhibiting it at higher (2–5 µM) concentrations [21].

## 5. SA Acts Mainly via the Regulation of Auxin Distribution in the Root

As already mentioned above, the influence of SA on adventitious rooting and root biomass is mediated via tight crosstalk with auxin. In this section, we overview the molecular aspects of the interplay between these hormones in roots.

**Figure 2:**
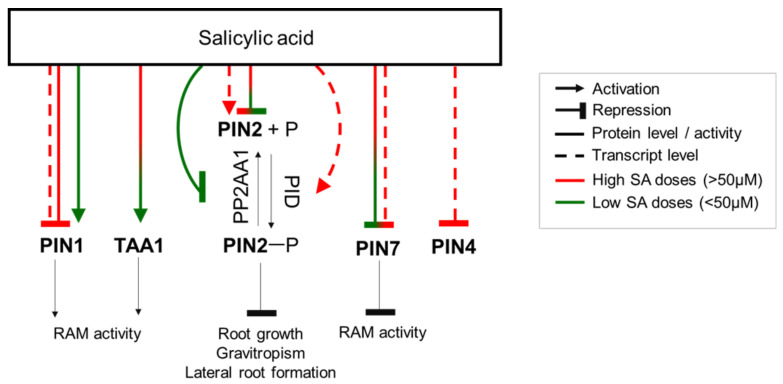
SA regulates auxin distribution in *Arabidopsis thaliana* root. An SA treatment activates the auxin biosynthetic enzyme TAA1 and inhibits the PIN2/PIN7 auxin efflux carriers. Low doses of SA activate PIN1, promoting auxin accumulation and transportation, which leads to a distal meristem extension. High doses of SA decrease PIN1 expression, inhibiting meristem activity. Moreover, SA elevates PIN2 phosphorylation, thereby affecting auxin transport. TAA1, TRP AMINOTRANSFERASE OF ARABIDOPSIS 1; PIN1/2/4/7, PIN-FORMED 1/2/4/7; PP2AA1, Protein Phosphatase 2A subunit A; PID, Protein kinase PINOID; P, phosphate; RAM, root apical meristem.

In Arabidopsis, SA differentially regulates the protein level of PIN-FORMED (PIN) auxin transporters in a concentration-dependent manner. Both low (30 µM) and high (150 µM) SA concentrations reduce PIN2 and PIN7 levels, while only a high SA concentration decreases PIN1 by 40%, whereas a low concentration of SA elevates PIN1 by 30% [25]. It is worth noting that, in contrast to the protein level, a high SA dose (250 µM) increases the transcript number for *PIN2*, though it still reduces the number of *PIN1*, *PIN4,* and *PIN7* transcripts [9] (Figure 2). The Ser/Thr kinase PID and protein phosphatase PP2A, which carry out phosphorylation and dephosphorylation of PIN proteins, respectively, control their polar localization at the plasma membrane and thereby affect auxin distribution [203]. An SA treatment at a high concentration (250 µM) activates *PID*. In addition, SA is capable of binding to the A subunits of PP2A and suppressing its activity [26]. Increased PIN phosphorylation and decreased dephosphorylation result in a disturbance of auxin transport and gradients, which may explain some root phenotypes after an SA treatment (Figure 2). Thus, PIN2 hyperphosphorylation occurs 15 min after an SA treatment (40 µM) and becomes more pronounced 45 min later, contributing to a reduction in primary root growth, lateral root formation, and the gravitropic response [26]. SA (25–50 µM) also affects PIN proteins by repression of their endocytosis [129,204]. A higher SA concentration (100 µM) and more prolonged treatment (24 h) result in the condensation of PIN2 proteins into hyperclusters on the cell surface, hampering auxin transport and impairing root gravitropism [205].

SA can also alter PIN2-based polar auxin transport in the root via a cGTPase NITRIC OXIDE-ASSOCIATED PROTEIN1 (AtNOA1) [206]. This pathway likely contributes to the regulation of root waving in Arabidopsis, which is induced by an SA treatment (with the maximal amplitude at 30 μM within a concentration range of up to 50 μM), in an NPR1-dependent manner. Besides auxin transport, AtNOA1 mediates an SA-induced cytosolic Ca^2+^ increase, which is crucial for SA-induced root waving. AtNOA1 expression is activated in the root after an SA treatment. Despite the fact that AtNOA1 mediates NO production during plant development [207,208] and SA induces nitric oxide (NO) production in roots [209], the function of AtNOA1 in SA-induced root waving is independent of NO [206].

Along with the modulation of auxin transport, an SA treatment may enhance IAA content in the root. The expression of TRP AMINOTRANSFERASE OF ARABIDOPSIS 1 (TAA1), the first enzyme in the main pathway of auxin biosynthesis, increases three-fold in root tips exposed to SA (both 30 µM and 150µM) [25]. Arabidopsis plants overexpressing the *GH3.5/WES1* gene, encoding an auxin-conjugating enzyme (IAA-amido synthetase), have a decreased free IAA level, increased SA content, a reduced number of lateral roots [199], and a reduced primary root length [210]. GH3.5/WES1 is induced by IAA, ABA, and SA, which highlights that not only hormone biosynthesis but also other aspects of hormone metabolism participate in SA–auxin crosstalk.

The complex interactions between SA and IAA differentially influence root architecture. In maize, IAA treatment (1 µM) decreases overall root biomass and root length, while SA (1.5 µM) increases both characteristics [42]. Attacks of *Diabrotica virgifera* larvae, which are soil-borne pathogens, seriously damages roots, causing an increase in IAA and SA in different parts of the maize root tip (in the distal and proximal regions, respectively). SA’s effect on auxin signaling in roots is concentration-dependent [25]. In Arabidopsis, low (30 µM) and high (150 µM) SA concentrations increase and decrease the activity of the DR5:GFP auxin sensor, respectively. It has been reported that 0.5 mM SA blocks strong induction of DR5:GUS by 1 µM 1-naphthaleneacetic acid (NAA), a synthetic auxin, in Arabidopsis roots [151]. Auxin and SA signaling antagonize each other in the regulation of lateral root initiation (see below).

## 6. SA Regulates Columella Development

The root stem cell niche (SCN) consists of the mitotically inactive SCN organizer and the quiescent center (QC) and is surrounded by stem cells, including cortex/endodermis initials (CEIs), stele cells initials (SCIs), epidermal/lateral root cap initials, and columella stem cells (CSCs) [211,212] (reviewed in [213]). Lateral root cap initials and CSCs form the root cap or distal meristem, and the rest of the stem cells, together with their dividing descendants, belong to the proximal meristem (reviewed in [214]). In the event of stem cell damage, QC cells can divide to replenish them. Ablation of the QC cells causes the precocious differentiation of CSCs, determined by starch accumulation.

Both an SA treatment (10–30 µM) and an elevation of endogenous SA levels contribute to increases in the frequency of QC cells division, which are normally relatively mitotically inactive. Elevated SA levels give rise to additional QC cells with decreased levels of WUSCHEL RELATED HOMEOBOX 5 (WOX5), the QC marker [25,215]. In SA over accumulating *constitutively activated cell death 1* (*cad1*) mutants [216] and in wild-type plants treated with a low SA concentration (10 µM) for 5 days, CSCs prematurely differentiate into columella [215]. A high percentage of plants (40–70%) do not have the CSC layer between the QC and the differentiated columella cells. In contrast, treatment with a slightly higher SA concentration (30 µM, for 5 days), results in the formation of two to four extra tiers of QC/CSC-like cells, which express both QC (WOX5) and CSC (J2341) markers and which lack starch granules [25]. Enlargement of the distal meristem is also reported in the SA-accumulating mutants *dnd1* and *dnd2*. A further increase in the SA concentration (150 µM, for 5 days) does not affect the distal meristem organization compared to the wild type aside from the bigger size of the columella cells and the lack of starch granules.

These alterations in the distal meristem occur via at least two mechanisms: changes in ROS level and in auxin distribution. SA signaling promotes ROS production and homeostasis [12,215]. ROS signaling regulates QC cell division and CSC differentiation [187,215,217]. Upon a low-dose SA treatment (30 µM, for 24 h), auxin rises in the Arabidopsis root tip by increasing auxin biosynthesis and the PIN1 level, as well as by suppressing PIN2 and PIN7 [25]. Higher SA concentrations (100–150 µM, for 24 h) [25,205], or prolonged treatment with low-concentration SA (10–30 µM, for 5 days) [215], reduces auxin levels according to decreased DR5 auxin sensor activity in the QC and CSCs. High SA levels (150 and 250 µM) suppress auxin flow from the stem to the root tip, mainly due to a decrease of PIN1 [9,25]. Thus, low SA doses increase auxin levels in CSCs and promote their differentiation, while high SA doses decrease auxin levels and retard CSC differentiation.

Experiments with plants treated with auxin, possessing excessive auxin accumulation and with auxin-deficient mutants, confirm the role of auxin in SA-induced columella changes. Seedlings, treated with NAA (1–5 µM) and PIN-dependent auxin transport inhibitor 1-N-naphthylphthalamic acid (NPA; 0.05–5 µM), as well as transgenic lines with increased endogenous auxin levels, also lose the CSC layer in their root tips due to precocious CSC differentiation [218]. In contrast, mutants defective in auxin biosynthesis, signaling, or PIN auxin efflux in the columella, have several layers of CSCs. Thus, facilitating CSC differentiation, very low SA doses imitate the effects of auxin treatment or disturbance of auxin transport, whereas increased SA doses, which result in several CSC layers, phenocopy mutants in auxin biosynthesis and signaling.

It should be noted that a variety of other hormones play an important role in the control of QC cell division and CSC differentiation. Elevated levels of endogenous ethylene or an ACC treatment (50 µM) also cause extra cell divisions in QC and the formation of several additional cells with QC identity [219]. Inhibition of ethylene biosynthesis by 2-aminoethoxyvinyl glycine (AVG) in wild-type plants reduces the number of columella layers. JA-treated roots (20 µM, 2 days and more) also have extra cells with QC identity, and this effect is ethylene independent [170]. In parallel, JA destroys CSC identity; CSC marker J2341 is expressed in more than one layer but at the same time, some cells below the QC have starch granules. Brassinosteroids at low concentrations (1 fM–0.1 nM) stimulate mitotic reactivation of QC cells and inhibit CSC differentiation, whereas higher concentrations (4 nM) promote CSC differentiation up to the disappearance of the CSC layer [149,220]. In the QC, ABA antagonizes SA and promotes QC quiescence. QC divisions, producing extra QC cells, were detected after blocking ABA biosynthesis and in ABA deficient or ABA insensitive mutants [33]. ABA regulation of QC divisions is ethylene independent. In both proximal and distal meristems, ABA suppresses cell differentiation. ABA (50–500 nM) increases the length of the proximal meristem without activating cell divisions, just by increasing the number of undifferentiated stem cell descendants. In the distal meristem, blocking ABA biosynthesis results in starch accumulation, not only in CSC but also in QC cells. ABA suppression of cell differentiation in the distal meristem depends on the WOX5 and AUXIN RESPONSE FACTOR 5/MONOPTEROS (ARF5/MP) function. Thus, SA (10–30 µM) stimulates QC divisions similar to ACC (50 µM), JA (20 µM), and brassinosteroids (1 fM–0.1 nM), and unlike ABA [33,149,170,219,220]. In turn and similar to very low SA doses, treatment with auxin or 4 nM brassinolide promotes CSC differentiation. Similar to higher SA doses, lower auxin and brassinolide concentrations, as well as ABA treatment, inhibit CSC differentiation and thereby elevate the number of CSC layers [25,33,149,215,218,220]. In the proximal meristem, where SA inhibits cell divisions, ABA suppresses cell differentiation [25,33]. Although there likely exists a complex interplay between SA and the signaling pathways of other hormones, the detailed mechanistic insight into this crosstalk requires further investigation.

The prolonged (3–5 days) treatment of 3–4 DAG seedlings with SA (30 µM) also enlarges the distal meristem width by disturbing cell division planes and increasing division frequency of epidermal/lateral root cap initials and CEIs [25]. Normally the QC cells with increased division rates and the CEIs with disordered divisions begin to form in the roots of older seedlings, starting from 10 DAG, where this phenotype is observed in about 40% of plants. In *Catharanthus roseus* hairy root tissue cultures obtained from *Agrobacterium rhizogenes* infected leaves, SA at a very low concentration (10 fM) also expands the root cap width and increases the number of columella cells [189].

## 7. SA Controls Radial Root Patterning

SA (30 µM) changes radial root patterning in the epidermis and subepidermal tissues in *A. thaliana* [25]. Multiple extra divisions are detected in the cells of these outer layers in SA-treated 3–5 DAG seedlings, starting from 36 h of treatment. The divisions are radially (tangentially) oriented in the epidermis, cortex, and endodermis, leading to the formation of extra cells or cell files in these tissues. Additionally, an SA treatment (10–30 µM) induces periclinal divisions in the endodermis resulting in the formation of the middle cortex, an intercalary tissue between the endodermis and the cortex [21,25]. Normally, the middle cortex starts forming in the roots of older seedlings (10–14 DAG) [25,221,222].

The low SA concentration induces middle cortex formation using two aforementioned mechanisms involved in SA-induced changes in the distal meristem: SA increases auxin accumulation [25] and promotes hydrogen peroxide production via repressing catalases [21]. Accordingly, the middle cortex develops prematurely after a hydrogen peroxide treatment [223]. SHORT-ROOT (SHR) and CYCD6;1 regulate middle cortex formation [25,222]. SA (30 µM) decreases SHR in endodermis cells and thereby activates CYCD6;1 and enlarges the CYCD6;1 expression domain to include the cortex and endodermis [25]. This results in middle cortex formation from endodermis and tangential cell divisions in some endodermis, cortex, and epidermis cells. In addition, SHR elevates reactive oxygen species, mainly hydrogen peroxide, the scavenging of which greatly reduces SHR mediated periclinal divisions [21]. Furthermore, SHR promotes hydrogen peroxide generation by activating the SA pathway.

It is not clear if other pathways contribute to SA-induced formation of the middle cortex. Although SCARECROW (SCR) is not an SA target [25], *scr* mutants prematurely form the middle cortex at phloem poles (starting at 3 DAG and being extensive by 7 DAG) indicating that SCR regulates both the timing and location of middle cortex development [222]. SHR protein is synthesized in the central vascular cylinder and moves into an adjacent endodermis, where SCR blocks further SHR movement [224]. In contrast to SA, GA and ABA suppress middle cortex development, and their interaction in this process is complex [222,225,226,227]. SCARECROW-Like 3 (SCL3), a direct target of SHR and SCR, controls middle cortex formation, downstream of DELLA, in the GA signaling pathway [228]. SEUSS is the upstream regulator of SHR, SCL, and SCL3 during middle cortex development, integrating SHR and GA pathways [229]. GA downregulates both SCL3 and SEUSS [228,229]. GA- AND ABA-RESPONSIVE ZINC FINGER (GAZ), which is repressed by both ABA and GA, affects in feedback the metabolism of both hormones and thereby the timing of middle cortex formation [227]. Ethylene, like SA, promotes middle cortex development but the underlying mechanism is unknown [226].

The endodermis can produce several cortex layers while maintaining a single endodermis layer. For example, in white mustard, which belongs to the Brassicaceae family, as does Arabidopsis, there are four concentric rings of cortical cells, organized in such a way that the cells of each ring are located opposite each other, forming radial intercellular spaces from the endodermis to the epidermis, whereas in tomato, in five cortex layers, the cell positions in the concentric rings are alternate [230]. In maize roots, five–six concentric cortex rings can be subdivided into the inner, with opposite, and the outer, with alternate, cell arrangements. It would be interesting to investigate if SA plays any role in these cortex arrangements.

## 8. SA Alleviates Changes in Root System Morphology Induced by Abiotic Stresses

Due to the sessile nature of plants, roots react to various abiotic stresses, changing the root system architecture. Treatment with SA completely removes the consequences of weak and moderate stresses and partly recovers inhibition of root growth caused by severe stresses (Table S3). For example, SA (10 μM) application to chickpea plants exposed to weak or moderate and strong cadmium (Cd) stress increases, restores and partly restores root length, respectively [231]. In some cases, SA’s protective effect is due to it reducing stress factor toxicity [232,233,234] (reviewed in [235]). For example, the application of exogenous SA elevates aluminum (Al)-induced citrate efflux from the roots of *Cassia tora*, which is associated with an increased tolerance of seedlings to Al [44]. In the present review, we consider only those cases where SA treatments protect plants from damage to the root system caused by abiotic stresses.

SA priming of seeds (soaking seeds in an SA solution and subsequent drying) either completely or partially recover seed germination inhibited by salinity, drought, and Cd stresses [71,79,82,236] (Table S3). The effective SA concentration depends on plant species and stress severity. SA priming of sesame (70–350 μM) and wheat (15 μM) seeds mitigated salinity-induced (40–50 mM NaCl) inhibition of seed germination [71,97]. SA priming of mungbean (0.01 μM), fenugreek (15 μM), Arabidopsis (50–500 μM), wheat (0.3–1 mM), and barley (1 mM) seeds alleviated even higher salinity levels (100 mM NaCl and more) [72,75,77,114,122,237]. SA seed priming, the addition of SA to the soil or growth medium, and even spraying shoots with SA can rescue root length and biomass, suppressed by salt, drought, chilling, nickel, cadmium, arsenic, silicon, zinc, and lead stress (Table S3).

For several species, low SA concentrations (*Carum copticum*, 10 nM; Arabidopsis, 100 μM; and *Limonium bicolor*, 0.08–0.2 mM) rescue salinity-induced inhibition of seed germination, while higher doses (*Carum copticum*, 1 μM–10 mM; Arabidopsis, 500 μM–1 mM; and *Limonium bicolor*, 0.24–0.28 mM) enhance it [74,78,238]. In Arabidopsis, low SA concentrations are not only effective in increasing root growth but also decrease K^+^ leakage from cells due to acute salt stress, whereas high SA concentrations not only inhibit root growth but also have no impact on K^+^ leakage [25,239]. Both NaCl-induced K^+^ leakage and H^+^ influx are most strongly decreased in roots treated with low SA concentrations (10–50 μM) [239]. In most cases, those SA concentrations, which enhance or at least do not influence root growth under normal conditions, promote plant recovery from stress conditions [231,236,240] (Table S3). This recovery is related to SA’s protection of cell divisions. The SA treatment of wheat seeds increases the mitotic index in the root apical meristem and thereby promotes root tolerance to high salinity and their enhanced recovery after stress [143].

SA protection of the root system under stress conditions is also related to the optimal SA endogenous level. For example, soil alkalinity, one of the threats to crop productivity, decreases both tomato root length and dry weight [56]. Treatment with SA (100 μM), not only compensates for this damage but additionally enhances both parameters compared to control plants. Soil alkalinity elevates the endogenous SA level in roots, whereas simultaneous SA treatment completely eliminates this increase.

In roots, the alleviation of abiotic stress damage with SA occurs via SA crosstalk with other hormones and via hydrogen peroxide and nitric oxide produced in response to both SA and stress [241,242,243,244] (reviewed in [235]). We have already discussed SA involvement in crosstalk with hormones, ROS, and nitric oxide, however, stress responses have some specific characteristics. For example, in Arabidopsis roots, SA and ethylene signaling interact with each other in response to Al [245]. Single *npr1* and *ein2* mutants have a lower decrease in root fresh weight compared to the wild type and *npr1 ein2* double mutant. SA-mediated stress responses may recruit other hormonal pathways. In barley exposed to Cd (15 µM), IAA content in root tips increases three-fold [243]. Both Cd and IAA (1 µM) treatments result in root growth suppression and swelling. Post-treatment with SA (0.25–0.5 mM) rescues the normal root phenotype without affecting IAA content in roots, possibly acting on IAA signaling pathways. Elevation of auxin and ethylene signaling follows iron deficiency-induced SA accumulation in Arabidopsis [54]. An increase in endogenous ABA levels is a possible intermediate in SA protection against various abiotic stresses [233,246].

## 9. SA Couples Root Morphology and Plant–Soil Biota Interactions

Due to their direct contact with the soil, roots are vulnerable to soil-borne pathogens such as fungi, bacteria, viruses, nematodes, and herbivorous insects. Recent studies demonstrate that some SA-induced morphological and morphogenetic changes are a part of the strategy that SA utilizes to restrict pathogen invasion in the root. Some pathogens, for example, viruses, are capable of invading epidermal and cortical cells intracellularly through plasmodesmata [247,248,249]. In Arabidopsis, exogenous SA (50 μM, 24 h) or cucumber mosaic virus-induced SA, triggers plasmodesmal closure in root meristematic cells via Remorin-dependent membrane lipid organization to impede virus spread [250]. This regulation employs NPR-mediated SA signaling. In asparagus, SA pretreatment facilitates *Fusarium oxysporum*-induced cell wall reinforcement in the root due to enhanced lignin synthesis, thereby alleviating pathogen propagation [251]. SA antagonizes auxin in lateral root formation to restrict bacterial infection. *Pseudomonas syringae* strain *Pto* DC3000 invades Arabidopsis plants through emerged lateral roots and then induces lateral root formation by producing auxin, which activates the ARF7/ARF19–LATERAL ORGAN BOUNDARIES-DOMAIN 16 (LBD16)/LBD18 regulatory module [188,252]. SA represses lateral root formation via the induction of *PATHOGENESIS-RELATED GENE 1* (*PR1*) and *PR2* transcription, thereby decreasing the number of potential pathogen entry sites [188]. Notably, the bacteria fight against this defense strategy; auxin-activated ARF7 directly represses the transcription of *PR1* and *PR2* to derepress lateral root development. In response to pathogen attacks, plants accumulate SA, which represses auxin signaling [151] and transport [9,25,253].

In addition to pathogens, plant roots contact a plethora of non-pathogenic soil microorganisms. The microbial community associated with the plant roots facilitates physiological and morphological functions of roots, including organogenesis and root architecture (reviewed in [254,255,256]). SA can impact these processes by shaping root microbiota (reviewed in [257,258]). An essential role of SA in modulating colonization of the root by specific bacterial families was nicely demonstrated for Arabidopsis [35]. Presumably, SA functions as a part of the immune system or affects microbe—microbe interactions and root physiology via yet undefined mechanisms.

In legumes, symbiotic Rhizobium bacteria under nitrogen-limiting conditions, trigger the plant-guided formation of novel root organs called nodules, which promote rhizobia-mediated nitrogen fixation from the atmosphere (reviewed in [254,259]). The symbionts are capable of escaping host immunity to invade the root (reviewed in [260]). It is generally accepted that exogenous SA (25–100 μM) inhibits the association of rhizobia with the host plant root and suppresses nodulation in indeterminate-nodule-type plants [261,262]. Decreased SA levels through the overexpression of *NahG*, promote infection thread and nodulation, including the determinate-nodule-type plant *Lotus japonicus* [14]. The bacterial Nod factor contributes to the avoidance of microbe-induced SA accumulation in the roots of at least some species, such as peas and alfalfa, while in others (for example, vetch) this tolerance does not depend on Nod [261,262,263]. Interestingly, low exogenous SA concentrations of 5–10 μM, stimulate nodulation in several legume species including the determinate-nodule-type plant *L. japonicus* [262,264]. This gives an idea of a possible SA contribution in the nodulation process, independent of plant immunity mechanisms. This is consistent with the recently described role of SA in the regulation of the subcellular localization of plasma membrane microdomains, which is essential during the early stage of nodulation in soybeans [265].

Another example of SA participation in symbiont-induced morphogenesis is the induction of second-order lateral root development in Arabidopsis, elicited by the rhizobacterium *Serratia marcescens*, strain 90–166. It is alleviated in SA-deficient plants overexpressing *NahG*, highlighting the impact of the SA signaling pathway in this morphological trait [266].

## 10. Conclusions: SA Links Stress Response and Development

Salicylic acid is often considered in the context of its protective mechanisms against biotic and abiotic stress. Providing this dual function, SA greatly influences root development starting at seed germination, through to root elongation, root branching, and adventitious rooting. Treatment with SA often causes dwarf plant phenotypes, which is interpreted as being an SA-induced shift of plant resources from growth to defense. To ensure optimal plant growth in an unfavorable environment, different mechanisms controlling the growth-defense balance in plants have evolved, in which SA plays an important role. First, SA-mediated concentration-dependent positive/negative feedback regulation of its own biosynthesis fine-tunes this balance [63]. Another vivid example is the growth and stress regulator CPR5, which attenuates both (1) SA levels, and (2) SA- and the endoplasmic reticulum stress-induced IRE1-bZIP60 arm [11]. The latter is capable of promoting inhibition of root elongation under elevated SA conditions and mediating the unfolded protein response. Therefore, CPR5 directly manages the trade-off between plant growth and stress responses. Being the precursor of both IAA and SA, shikimate may act as a switch from plant development to a protection mode (reviewed in [267]). Thus, intense morphogenesis, requiring local IAA biosynthesis, limits the ability of a proper response to stress, and stress response blocks morphogenesis.

On closer inspection, SA-mediated morphological changes such as the reduction of lateral root formation, enhanced adventitious rooting, nodulation, cell wall lignification, and plasmodesmal closure, can directly impede pathogen invasion in the root or facilitate plant growth under adverse conditions. In this context, it is important to highlight that most of the SA effects are concentration-dependent, which was demonstrated at least in several species. Consequently, there are two bioactive concentration windows for SA in the root system; at low levels, it acts as a developmental regulator, and at high levels, SA acts as a stress hormone [25]. It has been suggested that endogenous SA is a hormetic regulator which produced heterosis in Arabidopsis Columbia/C24 hybrids at sub- and supra-optimal doses [66]. Most of the evidence reviewed herein, suggests that SA’s hormetic abilities act to stimulate growth at low doses and to inhibit growth at high doses. 

## Figures and Tables

**Table 1:** The influence of biotic and abiotic stress factors on SA content in roots.

Plant Species	Stress Factor Type	Stress Factor ^1^	SA Level	Reference
Biotic stress				
*Cucumus sativus* L.	Necrotrophic fungus	*Rhizoctonia solani*	↑	[37]
*Zea mays* L.	Root herbivore	*Diabrotica virgifera* larvae	↑	[42]
*Arabidopsis thaliana* L. (Bur-0)	Biotrophic protist	*Plasmodiophora brassicae*	↑	[43]
*Arabidopsis thaliana* L. (Col-0)	Biotrophic protist	*Plasmodiophora brassicae*	-	[43]
Abiotic stress				
*Cassia tora* L.	Aluminium	Al (10–50 µM)	↑ (RT)	[44]
*Glycine max* L.	Aluminium	AlCl_3_ (30 μM)	↑ (RT)	[45]
*Hordeum vulgare* L.	Heavy metal	CdCl_2_ (25 µM)	↑ (F)	[46]
*Triticum aestivum* L.	Heavy metal	Cd(NO_3_)_2_ (250 µM)	↑ (F)	[47]
*Arabidopsis thaliana* L. (Col)	Heavy metal	CdCl_2_ (50 μM)	↑	[48]
*Oryza sativa* L.	Chilling	5 °C	↑ (F + C)	[49]
*Cucumis sativus* L.	Chilling	8 °C	↑ (F + C)	[50]
*Hordeum spontaneum* L.	Drought	PEG 6000 (−0.75 to −1.5 MPa)	↑	[51]
*Hordeum vulgare* L.	Drought	PEG 6000 (−0.5 MPa)	↑	[52]
*Scutellaria baicalensis* Georgi	Drought	PEG 6000 (15%)	↓ (F + T)	[53]
*Scutellaria baicalensis* Georgi	Salt	NaCl (150 mM)	↑ (F + T)	[53]
*Hordeum vulgare* L.	UV-B radiation	UV-B (0.84 W m^−2^)	↑	[52]
*Arabidopsis thaliana* L. (Col-0)	Iron deficiency	–Fe (0 µM)	↑ (F)	[54]
*Gossypium hirsutum* L.	Nitrogen deficiency	–N (0 µM)	↑	[55]
*Solanum lycopersicum* L.	Alkalinity	pH 9.0 buffer	↑	[56]

^1^ Biotic stress factors are limited to soil-borne pathogens. PEG, polyethylene glycol; RT, root tip; F, free SA; C, conjugated SA; T, total SA; “↑”, increase; “↓”, decrease; “-”, no difference.

**Table 2:** Concentration-dependent effects of SA on germination.

Plant Species	TP ^1^	SA Concent-Ration	TD ^2^	Ref ^3^	Plant Species	TP ^1^	SA Concent-Ration	TD ^2^	Ref ^3^
SA Increased Germination					SA Decreased Germination				
*Daucus carota* H.	1	7 μM	24 h	[69]	*Daucus carota* H.	1	7 mM	24 h	[69]
*Cucumis sativus* L.	2	10–50 µM	2–14 d	[70]	*Cucumis sativus* L.	2	100 µM–0.5 mM	2–14 d	[70]
*Arabidopsis thaliana* L.	2	100 µM	2 d	[73]	*Arabidopsis thaliana* L.	1	250 μM–1 mM	24 h	[74]
					*Arabidopsis thaliana* L.	2	2.5–5 mM	70 h	[75]
*Triticum aestivum* L.	1	10–20 μM	6 h	[71]	*Triticum aestivum* L.	1	30 μM	6 h	[71]
*Triticum aestivum* L.	1	0.5 mM	24 h	[72]	*Triticum aestivum* L.	1	1 mM	24 h	[72]
*Zea mays* L.	3	0.5–1.5 mM	24 h	[76]	*Zea mays* L.	3	3–5 mM	24 h	[76]

^1^ Treatment Procedure (TP). SA was applied in 1—seed priming, 2—seed germination, 3—embryo culture medium. ^2^ Treatment Duration (TD). h, hours; d, days. ^3^ References (Ref).

**Table 3:** Concentration-dependent effects of SA on root growth.

Plant Species	TP ^1^	SA Concent-Ration	TD ^2^	Plant Species	TP ^1^	SA Concent-Ration	TD ^2^	Ref ^3^
SA Increased Root Growth				SA Decreased Root Growth				
*Trigonellafoenum-graceum* L.	2	5–10 μM	8 d	*Trigonellafoenum-graceum* L.	2	15 μM	24 h	[77]
*Cucumis sativus* L.	2	10–50 µM	2–14 d	*Cucumis sativus* L.	2	0.1–0.5 mM	2–14 d	[70]
*Lens culinaris* L.	1	0.1–0.5 mM		*Lens culinaris* L.	1	1 mM		[3]
*Vicia faba* L.	1	0.5 mM		*Vicia faba* L.	1	1 mM		[4]
*Pennisetum glaucum* L.	1	0.5 mM	2 d	*Pennisetum glaucum* L.	1	0.5–3 mM	2 d	[5]
				*Pennisetum glaucum* L.	1	2–3 mM	2 d	[5]
*Triticum aestivum* L.	1	10 μM	6 h	*Triticum aestivum* L.	1	30 µM	6 h	[71]

^1^ Treatment Procedure (TP). SA was applied in 1—seed priming, 2—seed germination. ^2^ Treatment Duration (TD). h, hours, d–days. ^3^ References (Ref).

**Table 4:** Concentration-dependent effects of SA on adventitious rooting.

Plant Species	TP ^1^	SA Concent-Ration	TD ^2^	Plant Species	TP ^1^	SA Concent-Ration	TD ^2^	Ref ^3^
SA Increased Adventitious Rooting				SA Decreased Adventitious Rooting				
*Arabidopsis thaliana* L.	1	3–50 µM	5 d	*Arabidopsis thaliana* L.	1	0.1–0.2 mM	5 d	[25]
*Rhododendron pulchrum* Sw.	2	100 µM	62 d	*Rhododendron pulchrum* Sw.	2	10 mM	62 d	[142]
*Vigna radiate* L.	3	0.2–0.6 mM	24 h	*Vigna radiate* L.	3	0.8 mM	24 h	[195]

^1^ Treatment Procedure (TP). SA was applied in 1—rooting medium seedlings, 2—rooting medium cutting, 3—rooting medium hypocotyl cutting. ^2^ Treatment Duration (TD). h, hours; d, days. ^3^ References (Ref).

## Supplementary Materials

The following supporting information can be downloaded at: https://www.mdpi.com/article/10.3390/ijms23042228/s1.
